# Electrocardiographic Predictors of Atrial Fibrillation

**DOI:** 10.3390/medsci11020030

**Published:** 2023-04-07

**Authors:** Panagiota Anna Chousou, Rahul Chattopadhyay, Vasiliki Tsampasian, Vassilios S. Vassiliou, Peter John Pugh

**Affiliations:** 1 Norwich Medical School, University of East Anglia, Norwich NR4 7TJ, UK; pachousou@hotmail.com (P.A.C.); rahul.c@doctors.org.uk (R.C.); 2 Addenbrookes Hospital, Cambridge University Hospitals NHS Foundation Trust, Cambridge CB2 0QQ, UK; peter.pugh@addenbrookes.nhs.uk; 3 Norfolk and Norwich University Hospital NHS Foundation Trust, Norwich NR4 7UY, UK

**Keywords:** atrial fibrillation, atrial parameters, ventricular parameters, prediction

## Abstract

Background: Atrial fibrillation (AF) is the most common pathological arrhythmia, and its complications lead to significant morbidity and mortality. However, patients with AF can often go undetected, especially if they are asymptomatic or have a low burden of paroxysms. Identification of those at high risk of AF development may help refine screening and management strategies. Methods: PubMed and Embase databases were systematically searched for studies looking at electrocardiographic predictors of AF from inception to August 2021. Results: A total of 115 studies were reported which examined a combination of atrial and ventricular parameters that could be electrocardiographic predictors of AF. Atrial predictors include conduction parameters, such as the PR interval, p-wave index and dispersion, and partial interatrial or advanced interatrial block, or morphological parameters, such as p-wave axis, amplitude and terminal force. Ventricular predictors include abnormalities in QRS amplitude, morphology or duration, QT interval duration, r-wave progression and ST segment, i.e., t-wave abnormalities. Conclusions: There has been significant interest in electrocardiographic prediction of AF, especially in populations at high risk of atrial AF, such as those with an embolic stroke of undetermined source. This review highlights the breadth of possible predictive parameters, and possible pathological bases for the predictive role of each parameter are proposed.

## 1. Introduction

Atrial fibrillation (AF) is a supraventricular arrhythmia characterized by uncoordinated atrial electrical activation leading to ineffective atrial contraction and is the most common sustained pathological cardiac arrhythmia [1].

The estimated prevalence of AF in adults is 2–4% with an expected rise of at least two-fold by 2060 [2]. The prevalence of AF varies with sex and increases significantly with age, with those aged 80 or older having an estimated prevalence of 10–17% [3]. The lifetime risk of AF was estimated to be 25%, but this has now increased to 37% among adults over the age of 55 [1].

Whilst not inherently considered a life-threatening arrhythmia, the hemodynamic and thromboembolic complications of AF can lead to significant morbidity and mortality. Individuals with AF have a five-fold increased risk of stroke, and about 30% of embolic strokes of undetermined source (ESUS) are attributed to AF [4,5,6]. Moreover, AF appears to potentiate the impact of individual conditions, with the presence of AF post myocardial infarction being associated with greater mortality with a hazard ratio (HR) of 3.37 (95% confidence interval [CI]: 3.37–4.21) [7]. Furthermore, greater fatality and morbidity post-stroke were also seen in the European Community Stroke Project, where 33% of AF patients died within three months compared to 20% of those without AF [8].

While permanent AF is straightforward to identify on an electrocardiogram, paroxysmal AF (pAF) is considerably more difficult, especially in asymptomatic individuals. However, the risk of thromboembolic complications is considered the same in both conditions [9]. Thus, the detection of pAF is just as important, but is difficult if it is asymptomatic or infrequent. The aim, therefore, is to identify patients either prior to the onset of AF or early after the first paroxysm (even if asymptomatic) and risk stratify even asymptomatic patients for future AF development.

Multiple studies have suggested that AF occurs in the context of both electrical and anatomical abnormalities of the atria [10,11,12]. The 12-lead surface electrocardiogram (ECG) represents an easy, non-invasive approach to identify parameters that may represent electro-anatomical abnormalities that may either predict future AF or represent a pre-AF phenotype.

Developing a primary prevention approach to AF by identifying high-risk patients could potentially help with early identification of AF and appropriate therapy initiation, thereby reducing hospitalizations, AF-associated stroke incidence and the associated healthcare costs.

There are a number of potential markers of risk of AF development, including demographic, co-morbidity, electrocardiographic and echocardiographic data. We wished to focus on the electrocardiographic predictors of AF development in this systematic review.

## 2. Methods

**Figure 1:**
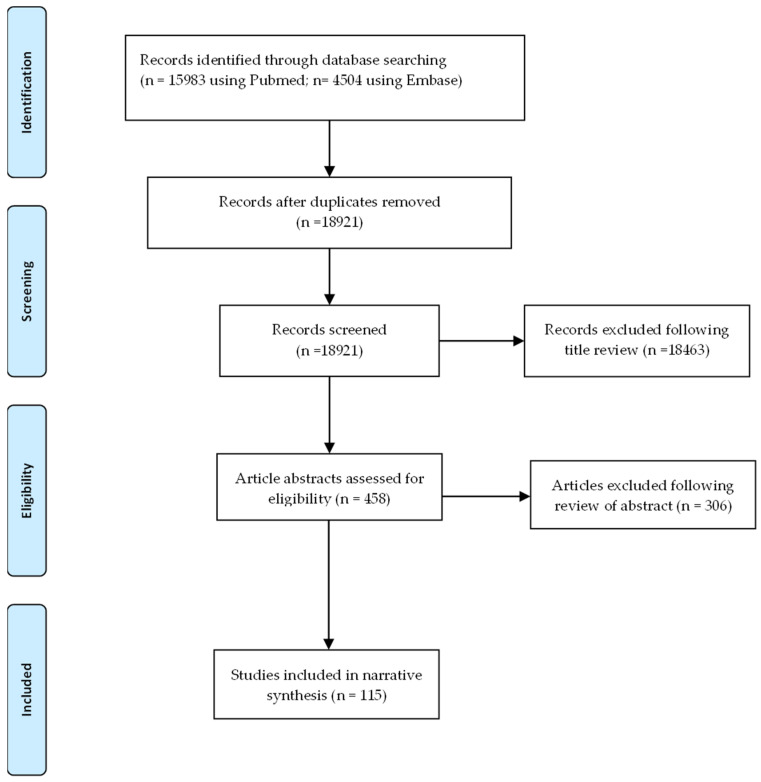
PRISMA flow diagram of the study selection process.

We searched PubMed and Embase using three different keyword strings encompassing ‘atrial fibrillation’, ‘AF’, ‘atrial flutter’, ‘electrocardiography’, and ‘predictor’ for articles from inception to 31 August 2021. Eighteen thousand nine hundred and twenty-one records were identified and independently screened. One thousand six hundred and thirty-nine were identified by title as being possibly relevant to the review and underwent review of the full text or abstract. A total of 132 full text articles were assessed for eligibility and 115 were included in this section (Figure 1). Articles without a clear definition of how AF was detected were excluded. Studies examining predictors of AF recurrence in the post-operative setting were also excluded. Additionally, studies looking at the prevalence or incidence of AF only were also excluded.

## 3. Results

The 12-lead surface ECG is helpful not only in the diagnosis of AF but also in identifying parameters associated with an increased predictive value for subsequent AF detection [13]. These parameters can be conveniently divided into those that are atrial (Table 1 and Table 2) and those that are ventricular (Table 3).

### 3.1. Atrial Indices

When considering atrial indices, they can be further divided into those that reflect conduction abnormalities, morphological abnormalities and mixed parameters.

#### 3.1.1. Atrial Conduction Parameters

##### P-Wave Duration and Partial Interatrial Block

P-wave duration represents the total time taken for a sinus impulse to propagate throughout the atria and is a surrogate for both intra- and interatrial conduction time. It is one of the most examined atrial indices, with respect to its predictive potential for AF. Prolongation of p-wave duration correlates with a slower conduction velocity within the atria, suggestive of atrial fibrosis, which could explain the association seen between prolonged p-wave duration and AF [77]. Differences in p-wave duration seen across leads can be a function of either differences in conduction velocities in different areas of the atria or of marked asymmetry of the atria themselves [41].

**Figure 2:**
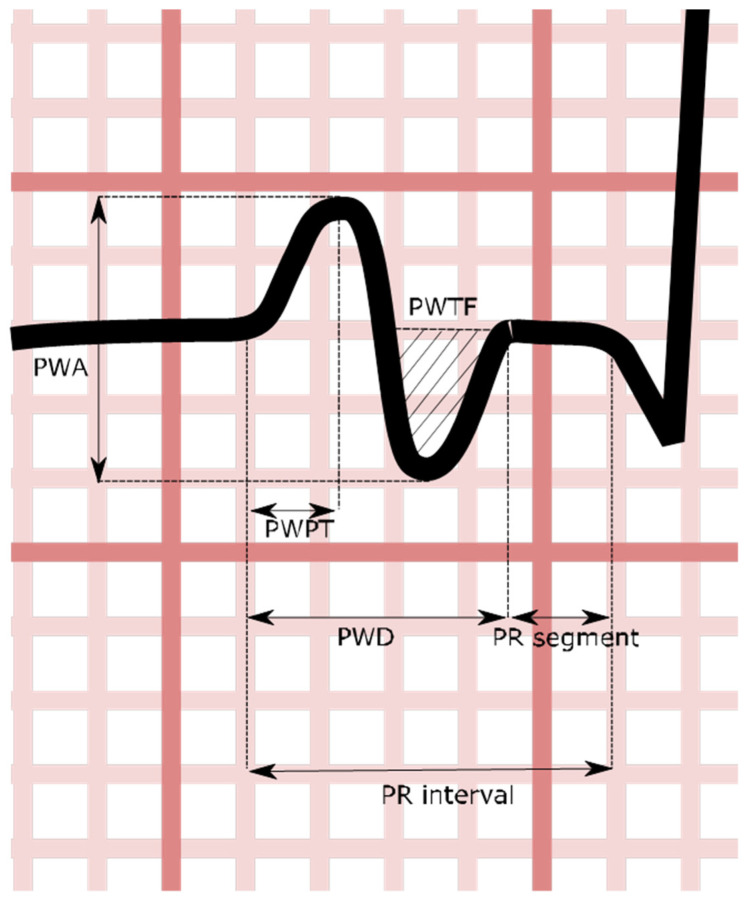
Stylized V1 p-wave demonstrating certain atrial indices. PWA: p-wave amplitude; PWPT: p-wave peak time; PWD: p-wave duration; PWTF: p-wave terminal force.

From a 12-lead ECG perspective, p-wave duration is measured from the first vertical deviation from the baseline (either upward or downward) to the return to baseline (Figure 2).

Partial interatrial block (P-IAB) is a parameter defined in the literature as a p-wave duration greater than 120 ms. It is thought to reflect the precursor state of atrial fibrosis [78]. In view of the overlap with p-wave duration studies, for the purposes of this review, partial interatrial block studies have been combined with p-wave duration studies.

The literature refers to different measures of p-wave duration, including the minimum, maximum and dichotomous cut offs and mean or median p-wave duration across the 12 leads (Table 1).

A total of 37 studies looked at the predictive potential of p-wave duration or P-IAB, with respect to subsequent AF detection (Table 1). Nine large cohort studies across general populations were conducted. Considering ESUS patients, there have been three studies that primarily examined this group, whilst one included ESUS patients. The other 24 were smaller studies.

Eight studies were case-control studies. In the non-case-control studies, the majority utilized either 12-lead ECG, Holter monitoring or medical record analysis for the identification of the development of AF. Only three studies used any form of continuous monitoring, two used device-based intracardiac electrograms (EGM) [38,45] and one used an ILR-based study [14].

Across the nine large cohorts, eight showed an association between PR duration ≥ 120 ms and the subsequent detection of AF. The one study that did not replicate this finding was based on the PROSPER study for older adults, which considered p-wave duration as a continuous variable and did not demonstrate a statistically significant increase in the risk of AF for 20 ms incremental increases in p-wave duration [39].

Of the four studies that looked specifically at ESUS, no statistically significant risk ratio was demonstrated.

If studies that used a dichotomous cut off of 120 ms are considered, ten out of the fourteen studies suggested a predictive role for PR > 120 ms, detecting subsequent AF. If a more prolonged duration is considered, there appears to be a stronger predictive role, with Nielsen et al.’s analysis of the Copenhagen ECG study suggesting an odds ratio (OR) of 2.06 compared to 1.50 and with PR durations of >130 ms rather than >120 ms [30], whilst Edenborn’s assessment of heart failure with preserved EF (HfpEF) patients suggested an HR of 9.68 for p-wave durations over 175 ms [18].

If, instead, p-wave duration was considered a continuous variable, neither of the studies demonstrated a statistically significant risk statistic for either 10 ms or 20 ms increases in p-wave duration.

Only one study looked at short PR durations and their relationship with future AF development. This was the assessment of the Copenhagen ECG study. Here, p-wave duration < 89 milliseconds was found to have an HR of 1.60 (95% CI 1.41–1.81) for AF, compared to a reference group with a p-wave duration of 106–111 milliseconds [30].

##### Advanced Inter Atrial Block

Advanced interatrial block (A-IAB) further stratifies prolonged p-wave duration according to inferior lead p-wave morphology. It is defined as p-wave duration of ≥120 ms plus biphasic morphology in the inferior leads [78]. Pathological studies have related A-IAB to the presence of atrial fibrosis [79].

Fifteen studies investigated the association between the presence of A-IAB and AF (Table 1). Out of these, five studies looked at the presence of A-IAB or P-IAB and its association with AF. There were three large cohorts that investigated this association, namely two retrospective cohorts that included Finnish adults and primary care patients and a prospective cohort that involved ARIC participants [16,20,57]. One study specifically looked at patients with ESUS, whilst a different one included patients with ESUS, palpitations and syncope [14,51]. The remaining studies were small cohorts looking at specific sub-groups.

With regards to the methods of detection, three studies utilized prolonged continuous monitoring: one by ILR and the others by a pacemaker with different cut off values for AF duration, ranging from AHRE ≥ 30 s to >5 min. The rest used different methods, including ECG, Holter and the documentation of AF in medical records or according to ICD codes.

Data in the literature are largely consistent with regards to A-IAB. Out of the fifteen studies, thirteen reported that the presence of A-IAB was associated with AF, whilst only two studies failed to demonstrate such an association. The first one included 165 patients with chronic kidney disease (CKD) at stage 4 or 5, while the second one involved 240 healthy Italians aged 25–79 years [17,22].

The results from the three large cohorts were consistent, demonstrating a positive association between A-IAB and AF detection with an HR of up to 3.38 (95% CI 2.99–3.81) [16,20,57]. Similarly, among patients with ESUS, A-IAB appeared to be an independent predictor of AF [14,51].

Additionally, it worth noting that in 2018, Tse et al. conducted a meta-analysis to investigate whether IAB predicts new onset AF or AF recurrence. They included 16 studies and a total of 18,204 patients. They demonstrated that A-IAB was a significant predictor of new onset AF with a pooled HR of 2.58 (95% CI 1.35–4.96). However, the risk of new onset AF did not reach statistical significance for P-IAB [80].

##### Other P-Wave Duration Parameters

Perez et al. investigated the usefulness of p-wave index, defined as the standard deviation (SD) of p-wave durations. P-wave index > 35 ms was predictive of AF with an HR of 1.70 (95% CI 1.15–1.56) across a general population of over 40,000 individuals [41].

Two different groups examined the role of p-wave onset and p-wave peak. Both studies found a positive association with AF [25,60]. However, no association was seen between prolonged maximum p-wave peak and p-wave end [25].

##### P-Wave Dispersion

P-wave dispersion (PWD) represents the difference between the maximum and minimum p-wave duration on a 12-lead ECG. It is felt that different p-wave durations reflect regional delays in atrial depolarization and are a result of inhomogeneous and discontinuous atrial conduction due to anisotropic distribution of conduction between atrial myocardial fibers [81,82]. These regional delays may potentially act as a substrate for AF. Whilst less well studied than p-wave duration, there have been a number of smaller studies looking at its role in the development of AF.

Seventeen studies looked at PWD and its association with AF (Table 1), including the large cohort study by Perez et al., looking at over 40,000 patients who had had an ECG for any indication [41]. Two other studies specifically looked at patients with strokes, either acute ischemic strokes or ESUS [19,36]. The remaining studies were small cohorts looking at specific sub-groups or small case-control studies.

Two studies used prolonged continuous detection methods: one used an external loop recorder and the other used a pacemaker. The rest used a combination of inpatient monitoring, Holter monitoring, ECG and medical records and various criteria for AF duration. Six studies employed case-control methodologies, whereby patients were stratified by the presence of AF.

Thirteen studies reported that increased PWD was associated with AF, whilst four studies did not find an association. No studies suggested that increased PWD reduced the risk of AF.

The only large cohort study, which enrolled 42,571 patients, showed that a PWD > 80 ms had an HR of 1.95 (95% CI 1.70–2.30) when adjusted for age and sex, but not in multivariable analysis [41]. Considering patients from smaller cohorts, increased PWD demonstrated a positive association with AF in ten studies and no association in four. Both studies looking at stroke showed a positive association [19,36].

Of note, there was one study that reported an assessment of PWD, but on closer assessment of the text, the definition used was very different to that of the above-mentioned studies. PWD was defined as p-wave duration divided by p-wave vector magnitude (Pvm) (calculated by the square root of the sum of the squared p-wave magnitudes in leads V6, II and half of the p-wave amplitude in V2). This approach was based upon Kors’ quasi-orthogonal transformation [83]. They found that this parameter was associated with AF, with an HR of 2.02 (*p* = 0.010) [26].

##### PR Interval

The PR interval represents the time taken for an electrical impulse to be transmitted from the sinus node through the atrioventricular node to the Purkinje fibers. On the 12-lead ECG, this is measured from the time of p-wave onset to the initiation of the QRS segment. Both prolonged and short PR intervals have been associated with AF. Suspected degenerative alterations of the myocardium and the conduction system causing prolongation of PR interval [76] might explain the association between prolonged PR interval and AF, while the association of a short PR interval might be attributed to genetics, as both the genetic loci responsible for either shortening or prolonging the PR interval were associated with an increased risk of AF [84].

Twenty-one studies looked at the association between PR interval and AF (Table 1). Of these, 19 investigated the association between prolonged PR interval and AF, whilst two also investigated the association between short PR interval and AF. As described in Table 1, different groups used different measurements, including PR interval in lead II, maximum PR interval, median or mean PR interval across 12 leads. Ten studies looked at this association in large cohorts, three in ESUS, with the rest in subgroups.

With regards to the method of detection, only three studies used prolonged monitoring by an ILR with an AF duration cut off of 30 s. The remaining studies used different methods, mainly ECG, Holter monitoring and the documentation of AF in medical records or based on ICD codes.

Ten studies considered PR interval as a continuous variable and seven considered it to be dichotomous with a cut off value of 196 ms, defining a prolonged PR interval with values ranging from 120–129 ms for a short PR interval. The remaining four used PR interval as both a continuous and dichotomous variable.

Twelve studies showed that prolonged PR interval either as a continuous or dichotomous variable is associated with the development of AF. Seven studies failed to demonstrate such an association. Two studies showed different results according to whether PR interval was used as a continuous or dichotomous variable.

Considering participants from large cohorts, six showed a positive association between prolonged PR interval and AF, two did not show an association and two showed that increasing PR interval (per 1 SD) was associated with AF, but not when used as a dichotomous variable. Amongst studies in the ESUS population, two did not demonstrate any association between prolonged PR interval and AF, whilst data from CRYSTAL AF demonstrated that for every 10 ms increase in PR interval, the HR for AF detected by ILR was 1.30 (95% CI 1.20–1.40) [69]. Out of the eight studies examining different subgroups, such as patients with ESUS and chronic kidney disease (CKD), four showed a positive association, whilst four did not find a significant association.

Additionally, a meta-analysis performed in 2014 showed that amongst 328,932 individuals from prospective cohorts, prolonged PR interval was associated with AF with a pooled HR of 1.30 (95% CI 1.13–1.49) [85].

One small study looked at the utility of PR variation defined as PR interval maximum–minimum in patients with >100 supraventricular ectopics (SVEs) per day. Amongst 207 patients, greater PR variation was associated with AF detected by ECG or Holter monitor [67].

Two studies investigated the role of short PR interval in predicting AF. The Busselton Health Study showed a positive association for PR interval < 120 ms [73], whereas the Copenhagen ECG study showed this was only significant in women [75].

From the ARIC cohort, Smith et al. looked at the PR segment, defined as the time between the end of the p-wave and the start of the QRS complex, and found that PR segment prolongation was independently associated with subsequent AF detection [25].

#### 3.1.2. P-Wave Morphological Parameters

##### P-Wave Axis

P-wave axis, a routinely reported measure on ECG represents atrial electrical activity. Abnormalities in this parameter are reflective of atrial pathology and possibly associated with an increased risk of AF development [86]. Mechanical and metabolic insults to the atria induce remodeling and abnormal electrical conduction, which results in abnormal p-axis and ultimately leads to AF [87,88].

P-wave axis is one of the better studied morphological p-wave features. There have been six studies assessing the relationship between p-wave axis and AF, all of them suggesting a positive predictive role of the abnormal p-wave axis and the subsequent development of AF (Table 2). A recent meta-analysis identified a pooled risk ratio of 2.12 for abnormal p-wave axis, and future AF detection from a total of 78,222 patients [89].

There have been four large retrospective studies looking at the ARIC, CHS and ACCORD populations and a general population of patients undergoing ECG. All four of these studies demonstrated a positive relationship between p-wave axis and the development of AF. One study looked specifically at p-wave axis amongst the population of ESUS patients, in which an abnormal p-wave axis was associated with an OR of 3.31 (95% CI 1.49–7.35) for AF [19].

**Table 2:** Atrial morphological parameters predictive of atrial fibrillation.

Authors, Year	Population (Size)	Study Type	Parameter Definition	Result	AF Detection
***P-wave axis***					
Dhaliwal et al., 2020 [90]	ACCORD (8965)	Retrospective	0–75°—normal	HR 2.65 (95% CI 1.76–3.99)	ECG
Acampa et al., 2019 [19]	Cryptogenic stroke (222)	Prospective	0–74°—normal	OR 3.31 (95% CI 1.49–7.35)	7-day Holter
Maheshwari et al., 2017 [91]	ARIC population (15,102)	Retrospective	0–75°—normal	RR 2.34 (95% CI 2.12–2.58)	ECG, Medical records
Rangel, O’Neal, and Soliman, 2016 [88]	CHS (4272)	Retrospective	0–75°—normal	HR 1.17 (95% CI 1.03–1.33)	ECG, medical records
Hayashi et al., 2014 [65]	P-pulmonale (591)	Retrospective	<74°—normal	HR 2.55 (95% CI 1.20–5.41)	ECG
Perez et al., 2009 [41]	Patients that had an ECG for usual indications (42,751)	Retrospective	Not defined	HR 1.90 (95% CI 1.60–2.40)	ECG
***P-wave terminal force***					
Kreimer et al., 2021 [14]	ILR (366)	Retrospective	≤−4000 µV·ms	HR 5.30 (95% CI 3.25–8.64)	ILR AF ≥ 30 s
Lehtonen et al., 2018 [21]	Hypertensives (665)	Retrospective	≤−4 mV·ms	HR 0.85 (95% CI 0.66–1.09)	Medical records
Cortez et al., 2017 [26]	Ischemic stroke patients from LSR (n = 227)	Prospective	≥0.04 mm·s	HR 1.00 (95% CI 1.00–1.00)	ECG
Goda et al., 2017 [92]	Ischemic stroke (226)	Retrospective	Per 0.01 mm·s	OR 1.61 (95% CI 1.24–2.09)	Inpatient monitoring
Sugiyama et al., 2017 [93]	Acute ischemic stroke (105)	Prospective	Continuous	OR 1.46 (95% CI 1.02–2.08)	24 h Holter
Rasmussen et al., 2017 [94]	Copenhagen Holter study (678)	Prospective cohort	>4000	HR 0.86 (95% CI 0.52–1.41)	ECG, inpatient monitoring, medical records
Baturova et al., 2016 [95]	Ischemic stroke with (55) and without AF (110) (165)	Case control	>40 mm·ms	OR 4.04 (95% CI 1.34–12.14)	Case control
Magnani et al., 2015 [29]	FHS (3110) ARIC (8254)	Prospective cohort	>4000 μV·ms	HR 1.00; 95% CI 0.71–1.40 HR 1.56; 95% CI 1.24–2.00	Medical records
Francia et al., 2015 [31]	Hypertensive (88)	Case-control	Continuous	HR 1.03 (95% CI 0.91–1.15)	ECG, Holter
Kamel et al., 2014 [96]	45–84 (6751)	Prospective cohort	Per 1 SD	HR 1.11 (95% CI 1.03–1.21)	ECG
Eranti et al., 2014 [97]	Middle-aged subjects (35–41 years) (10,647)	Prospective	≥0.06 mm·s	HR 1.91 (95% CI 1.34–2.73)	Medical records
Nishi et al., 2013 [98]	Hemodialysis (299)	Retrospective	≥0.04 mm·s	HR 4.89 (95% CI 2.54–9.90)	ECG
Hayashi et al., 2014 [65]	P-pulmonale (591)	Retrospective	Med free + >77 µV·ms	HR 2.22 (95% CI 0.70–8.31)	ECG
Soliman et al., 2009 [40]	General population (15,429)	Prospective cohort	>95th percentile	HR 1.22 (95% CI 1.14–1.31)	ECG
***P-wave amplitude***					
Yoshizawa et al., 2014 [34]	General population (136)	Retrospective	II	*p* = 0.032 *p* = 0.001	ECG
Kreimer et al., 2021 [14]	Patients undergoing ILR for syncope, palpitations, ESUS ILR (366)	Retrospective	II < 0.1 mV	HR 2.11 (95% CI 1.30–3.44)	ILR
Altunkeser et al., 2003 [46]	Patients with structural heart disease and LAD ≤ 5.0 cm with AF (n = 37) and without AF (n = 38) (75)	Case control	P-wave amplitude max P-wave amplitude min P-wave dispersion (amplitude)	*p* < 0.001 NS in multivariable analysis *p* < 0.01	Case-control study
***Other morphological parameters***					
Lentz et al., 2019 [99]	Patients on ibrutinib (168)	Retrospective	(1) Lead II-bifid p-wave, with 40 ms between peaks for ≥ 2.5 mm wide ≥ 100 msec in duration, (2) Lead V1-biphasic p-wave with terminal portion ≥ 40 msec in duration or terminal portion ≥ 1 mm deep or (3) PR interval ≥ 200 msec (intra-atrial conduction delay)	HR 5.40 (95% CI 1–9–15.4)	ECG, medical records
Hayashi and Horie, 2015 [32]	Patients with biphasic p-wave in lead II (141)	Retrospective	Amplitude of initial p-wave portion in lead II ≥ 73 (μV) Amplitude of terminal p-wave portion in lead III ≥ 48 (μV) Duration of initial p-wave portion in lead III ≥ 71 (ms)	HR 1.22 (95% CI 0.50–2.88) HR 1.60 (95% CI 0.68–3.72) HR 2.90 (95% CI 1.16–7.11)	ECG
van Diepen et al., 2010 [100]	Patients on pexelizumab with (315) and without AF (315) (630)	Case-control	M-shaped, W-shaped, irregular or notched p-waves	OR 1.68 (95% CI 1.03–2.73)	Case control (ECG, medical records)
***Compound conduction and morphological parameters***					
Rasmussen et al., 2020 [15]	Copenhagen Holter study (632)	Retrospective	P-wave area/duration index	HR 2.80 (95% CI 1.64–4.79)	ECG, inpatient monitoring
Tse et al., 2020 [101]	Mitral stenosis (59)	Retrospective	Mean p-wave area in V3	OR 1.08 (95% CI 1.01–1.16)	2 ECGs (persistent or permanent AF)
Hellman et al., 2020 [17]	CKD 4/5–non-dialysis (165)	Prospective	PWD ≥ 120 ms in lead II ± > 1 biphasic p-waves in leads II, III or aVF; or duration of terminal negative portion of p-wave > 40 ms or depth of terminal negative portion of p-wave > 1 mm in lead V_1_	Not significant	ECG, 24 Holter
Soliman et al., 2009 [40]	ARIC participants (15,429)	Prospective cohort	Maximum p-wave area Mean p-wave area	HR 1.13 (95% CI 1.05–1.23) HR 1.11 (95% CI 1.02–1.20)	ECG
De Bacquer, Willekens, and De Backer, 2007 [102]	55–74 years old with AF (40) and age-matched and gender-matched controls (120)	Nested case control	Maximum p-wave duration and notched or deflected p-wave morphology	OR 13.4 (95% CI 3.30–46.60)	Case control

ARIC, atherosclerosis risk in communities; ACCORD, Action to Control Cardiovascular Risk in Diabetes; CHS, Canadian Health Study; CABG, coronary artery bypass graft; MADIT, multicenter automatic defibrillator implantation trial; PPM, permanent pacemaker; PWD, p-wave duration; SND, sinus node disease; RR; HR, hazard ratio; OR, odds ratio; IPN, interpeak notch.

##### P-Wave Terminal Force

P-wave terminal force (PTFV1) has garnered significant interest as a possible predictor of AF. PTFV1 is the duration of the terminal (negative) part of the p-wave in lead V1 multiplied by the depth. If the p-wave terminal part is positive, then the interval extending from the first notch to the wave end must be considered [103] Commonly, it is considered abnormal when it is greater than 0.04 μV·ms, which is considered a marker of LA abnormality or enlargement [103,104].

One of the most pertinent criticisms of its use came from Jaroszynski et al. [105], who argued that it was particularly susceptible to lead position variation.

PTFV1 has been examined in 16 separate primary studies (Table 2), 12 of which were summarized in Huang et al.’s 2020 meta-analysis. This demonstrated a pooled odds ratio of 1.39 (95% CI 1.08–1.79) [104].

Considering the sixteen primary studies, five of them did not demonstrate a significant predictive role of PTFV1 in the prediction of AF. Four studies examined PTFV1 specifically in ischemic stroke, all of them demonstrating a positive result.

Only one study utilized continuous ILR monitoring for AF identification, while the rest utilized a mix of ECG, Holter monitoring or patient records [14].

##### P-Wave Amplitude

P-wave amplitude refers to the height of the p-wave in different ECG leads. Different groups have assessed its role as a predictor of AF by considering p-wave amplitude in different leads.

There have been four studies looking at its utilization as a predictor for AF (Table 2), all of which have suggested that increased p-wave amplitude is associated with AF detection. Only one study demonstrated that maximum p-wave amplitude, but not minimum p-wave amplitude, was significant [46].

##### Other P-Wave Morphological Parameters

There have been other p-wave morphological parameters studied in three small cohorts, as described in Table 2. The parameters are varied and use composite measures based on the shape of the p-wave in different leads. The exact parameter definition in each paper is summarized in the table. These have shown promising results for the possible prediction of AF, but more research is required, especially in larger general populations.

##### Compound Conduction and Morphological Parameters

There have been a number of studies that have combined p-wave conduction and morphology parameters. Generally, these have been smaller studies looking at populations that include individuals with mitral stenosis and non-dialysis CKD4/5 patients; however, there was a larger study that looked at p-wave area across 15,429 patients. In the two studies that examined p-wave areas, the mean area in lead III, as well as the overall mean and maximal p-wave areas, have all shown promise as possible AF predictors.

### 3.2. Ventricular Parameters

It is conceptually more difficult to associate changes in ventricular electrocardiographic parameters with a pre-AF or AF risk phenotype. Nevertheless, a number of studies have shown relationships between specific parameters and the risk of developing AF (Table 3).

**Table 3:** Ventricular parameters predictive of atrial fibrillation.

Author (Year)	Population and Size	Study Type	Parameter Definition	Result	AF Detection
***Left Ventricular Hypertrophy***					
Lehtonen et al., 2018 [21]	Hypertensive (2665) Non-hypertensive (3148) (5813)	Retrospective	Sokolov criteria Cornell	HR 1.51 (95% CI 1.14–2.01) HR 1.26 (95% CI 0.94–1.69)	Medical records
Patel et al., 2017 [106]	CHS (4904)	Retrospective	Minnesota	HR 1.50 (95% CI 1.18–1.90)	ECG
Chrispin et al., 2014 [107]	MESA (4942)	Retrospective	Sokolov product Cornell Framingham adjusted Cornell Minnesota Lewis Gubner and Ungerleider Sokolow voltage Cornell product Romhilt-Estes Perugia	HR 1.83 (95% CI 1.06–3.14) HR 1.36 (95% CI 0.72–2.58) HR 1.36 (95% CI 0.76–2.58) HR 1.26 (95% CI 0.76–2.08) HR 0.72 (95% CI 0.47–1.11) HR 1.02 (95% CI 0.62–1.68) HR 1.37 (95% CI 0.92–2.07) HR 1.69 (95% CI 0.94–2.31) HR 1.48 (95% CI 0.64–3.39) HR 1.35 (95% CI 0.79–2.28)	Medical records
Knuiman et al., 2014 [73]	Busselton Health Study participants (4267)	Prospective	LVH Minnesota code	HR 0.33; 95% CI 0.08–1.33)	ICD codes
Macfarlane et al., 2011 [39]	Older patients on pravastatin (5804)	Retrospective	LVH Minnesota code Definite Probable Possible	HR 2.13 (95% CI 1.38–3.28) HR 2.21 (95% CI 1.49–3.28) HR 1.30 (95% CI 1.03–1.64)	ECG
Perez et al., 2009 [41]	Patients that had an ECG for usual indications (42,751)	Retrospective	LVH Romhilt Estes criteria	HR 1.30 (95% CI 1.00–1.70, *p* = 0.046)	ECG
Watanabe et al., 2006 [108]	Niigata study (63,386)	Retrospective	LVH Sokolov- Lyon criteria	OR 1.39 (95% CI 1.1–1.75)	ECG
***QT interval***					
Patel et al., 2018 [109]	CHS (4181)	Retrospective	Prolonged > 95th percentile Per 1-SD increase	HR 1.50 (95% CI 1.20–1.88 HR 1.07 (95% CI 1.01–1.13	ECG, medical records
Lehtonen et al., 2018 [21]	Hypertensive (2665) Non-hypertensive (3148) (5813)	Retrospective	1 SD increment in QTc (Bazzet’s) Prolonged QTc > 450 ms (men), >460 ms (women)	HR 1.11 (95% CI 1.01–1.22) HR 1.26 (95% CI 0.78–2.03)	ECG
Nguyen et al., 2016 [110]	CHS (4696)	Retrospective	Prolonged QTc (Framingham)	HR 2.50 (95% CI 1.40–4.30)	ECG, medical records
Baturova et al., 2016 [95]	Ischemic stroke patients with AF (55) and without AF (110) (165)	Retrospective	QTc (Bazzet’s)	NS in multivariable analysis	Case control
Hoshino et al., 2015 [111]	Stroke (972)	Retrospective	QTc (per 10 ms increase)	OR 1.41 (95% CI 1.24–1.61)	Inpatient monitoring, 24 h Holter
Baturova et al., 2015 [112]	Ischemic stroke with (454)	Retrospective	QTc (Bazzet’s)	NS in multivariable analysis	ECG, medical records
Hayashi et al., 2014 [65]	Patients with p-pulmonale (591)	Retrospective	QT interval > 353 ms	HR 0.89 (95% CI 0.34–2.31)	ECG
Shulman et al., 2015 [70]	African American, Hispanic and non- Hispanic white (n = 50870)	Retrospective	QTc (per 10 ms increase)	HR 1.00 (95% CI 1.00–1.01, *p* < 0.001)	ECG
Mandyam et al., 2013 [113]	ARIC (14,538) + CHS (4745) + 2396 (Health ABC)	Retrospective	10 ms increase in QTc (Framingham)	HR 2.05 (95% CI 1.42–2.96)	ECG, medical records
Nielsen et al., 2013 [114]	Copenhagen (281,277)	Retrospective	QTc ≤ 372 ms QTc ≥ 464 ms QTc ≥ 458 ms	HR 1.45 (95% CI 1.14–1.84) HR 1.44 (95% CI 1.24–1.66) HR 2.32 (95% CI 1.52–3.54)	Medical records
Macfarlane et al., 2011 [39]	Older patients on pravastatin (5804)	Retrospective	Prolonged QTc (Hodges) (per 30 ms increase)	HR 1.21 (95% CI 1.11–1.32)	ECG
***QRS duration***					
Patel et al., 2018 [109]	CHS (4181)	Retrospective	Prolonged Per 1-SD	HR 1.00 (95% CI 0.77–1.30) HR 0.99 (95% CI 0.94–1.06)	ECG, medical records
Aeschbacher et al., 2018 [115]	ARIC (15314)	Retrospective	QRS 100–119 ms QRS ≥ 120 ms Per 1-SD increase	HR 1.13 (95% CI 1.02–1.26) HR 1.35 (95% CI 1.08–1.68) HR 1.11 (95% CI 1.07–1.15)	ECG, medical records
Cortez et al., 2017 [26]	Ischemic stroke patients from LSR (227)	Prospective	QRS duration (continuous)	HR 1.01 (95% CI 1.00 to 1.02, *p* = 0.354)	ECG
Baturova et al., 2015 [112]	Ischemic stroke (454)	Retrospective	QRS duration (continuous)	HR 1.02 (95% CI 1.00–1.03)	ECG, medical records
Shulman et al., 2015 [70]	African American, Hispanic and non-Hispanic white (50,870)	Retrospective	QRS duration (per 10 ms increase)	HR 1.00 (95% CI 1.00–1.00; *p* = 0.092)	ECG
Macfarlane et al., 2011 [39]	Older patients on pravastatin (5804)	Retrospective	QRS (per 20 ms)	HR 1.07 (95% CI 0.98–1.16; *p* = 0.14)	ECG
El-Chami et al., 2010 [116]	ADVANCENT (25,268)	Retrospective	QRS duration (continuous)	OR 1.20 (95% CI 1.14–1.25)	Medical records
***LBBB, RBBB, LAFB***					
Uhm et al., 2020 [117]	Patients that had ECG (n = 107,838)	Retrospective	NIVCD ≥ 110 ms	HR 2.57 (95% CI 1.07–6.16)	ECG, medical records
Nguyen et al., 2016 [110]	CHS (4696)	Retrospective	LAFB	HR 2.10 (95% CI 1.10–3.90)	ECG, medical records
Frontera et al., 2015 [71]	ILR implanted for syncope or palpitations (n = 200)	Retrospective	LBBB	OR 1.05 (95% CI 0.18–4.70)	ILR AF > 30 s
Frontera et al., 2015 [71]	ILR for syncope or palpitations (n = 200)	Retrospective	RBBB iRBBB	OR 3.60 (95% CI 0.84–14.99) OR 9.04 (95% CI 1.40–10.24)	ILR AF > 30 s
Knuiman et al., 2014 [73]	Busselton Health Study participants (n = 4267)	Prospective	LBBB	HR 1.84 (95% CI 0.90–3.74)	ICD codes
Perez et al., 2009 [41]	42,751	Retrospective	LBBB	HR 1.70 (95% CI 1.20–2.50)	ECG
Watanabe et al., 2006 [108]	Niigata study (63,386)	Retrospective	LBBB	OR 0.98 (95% CI 0.13–7.23; *p* = 0.98)	ECG
Watanabe et al., 2006 [108]	Niigata study (63,386)	Retrospective	RBBB	OR 0.84 (95% CI 0.46–1.53)	ECG
***Fragmented QRS***					
Hellman et al., 2020 [17]	CKD 4/5—non-dialysis (165)	Prospective	Notched R or S wave or the presence of ≥1 additional r-waves (R’) or in the presence of a wide QRS complex (>120 ms), >2 notches in R or S waves in two contiguous leads corresponding to a myocardial region,	Not significant	ECG, 24 h Holter
Yesin et al., 2018 [61]	STEMI (171)	Prospective	Various RSR’ patterns	OR 3.24 (95% CI 1.02–10.25)	Inpatient monitoring
***Poor R- wave progression***					
Lehtonen et al., 2018 [21]	Hypertensive (2665) Non-hypertensive (3148) (5813)	Retrospective	Poor r-wave progression	HR 1.49 (95% CI 1.01–2.20)	ECG
***Frontal QRS-T angle***					
Jogu et al., 2017 [118]	CHS (4282)	Retrospective	>Sex specific 95th percentile Per 10° increase	HR 1.55 (95% CI 1.23–1.97) HR 1.03 (95% CI 1.01–1.05)	ECG, medical records
***ST-T segment abnormalities***					
Lehtonen et al., 2018 [21]	Hypertensive (2665) Non-hypertensive (3148) (5813)	Retrospective	Negative t-wave in I and V6 Positive t-wave in aVR	HR 2.10 (95% CI 1.40–3.13) HR 3.47 (95% CI 1.16–10.34)	ECG
Bachmann et al., 2016 [119]	Copenhagen ECG study (138,404)	Retrospective	T peak- T end lead V5 < 5th % (58–77 ms) lead V5 < 95th % (116–140 ms)	HR 1.18 (95% CI 1.06–1.32) HR 1.09 (95% CI 0.99–1.22)	Medical records
Macfarlane et al., 2011 [39]	Older patients on pravastatin (5804)	Retrospective	Minnesota code 5-1 or 5-2 Minnesota code 4-1 or 4-2 See Supplementary Table S3	HR 1.69 (95% CI 1.34–2.13) HR 1.70 (95% CI 1.32–2.20)	ECG
Watanabe et al., 2006 [108]	Niigata study (63,386)	Retrospective	Mild ST abnormality Severe ST abnormality	OR 1.66 (95% CI 1.13–2.43) OR 5.12 (95% CI 2.30–11.38)	ECG

AF, atrial fibrillation; ARIC, atherosclerosis risk in communities study; CHS, Canadian Health Study; CI, confidence interval; CKD, chronic kidney disease; CRT, cardiac resynchronization therapy; ECG, electrocardiogram; HF, heart failure; HR, hazard ratio; ILR, implantable loop recorder; LAFB, left anterior fascicular block; LBBB; left bundle branch block; MESA, multi-ethnic study of atherosclerosis; ms, millisecond; NIVCD, non-specific intraventricular conduction delay; NS, non-significant; OR, odds ratio; RBBB, right bundle branch block; RWP, r-wave progression; SD, standard deviation; STEMI, ST-elevation myocardial infarction.

#### 3.2.1. Left Ventricular Hypertrophy

Left ventricular hypertrophy (LVH) can be diagnosed from a 12-lead ECG. Different criteria exist and have been used in different studies (Supplementary Table S1).

There have been seven large cohorts assessing LVH and AF. A variety of ECG LVH scores have been assessed, but the most common ones are the Minnesota code, Sokolow–Lyon and Cornell criteria. There were five large cohort studies that assessed general populations and two large studies which used specifically older patient cohorts or hypertensive individuals.

With respect to the method of AF detection, all of the studies used a combination of ECG, or AF present on medical records or ICD codes.

Of the seven studies, four demonstrated a consistently positive predictive association between ECG-defined LVH and AF, with one not showing any association and two studies providing mixed results across the different LVH criteria.

#### 3.2.2. QT Interval

Congenital abnormalities of the QT interval (short or long QT syndrome) are known to be associated with a high incidence of AF [120,121]. QT interval corrected (QTc) can be calculated using the Bazett, Hodges, Framingham and Fridericia formula (Supplementary Table S2). The QT interval reflects cardiac ventricular repolarization. It has been thought that the QT interval might be a marker of cardiomyocyte refractoriness [110,122].

The QT interval has had a reasonable amount of interest as a possible predictor of AF, with eleven studies examining the relationship between QT interval and AF detection. Of the six large cohort studies, five demonstrated a positive risk ratio AF.

Within the ischemic stroke population, there has been one case-control study, and two cohort studies. Only one of the cohort studies demonstrated a predictive role of the QT interval, with Hoshino et al.’s analysis of 972 stroke patients suggesting an OR of 1.41 (95% CI 1.24–1.61).

One study assessed a short QT interval [75], which was also noted to have a statistically significant predictive role in AF development.

None of the studies utilized any form of continuous monitoring, with retrospective medical record analysis and ECG assessment being the techniques used.

Zhang et al. performed a meta-analysis and found that when Bazett correction was utilized alongside a dichotomous cut off, there was a statistically significant predictive role, with a pooled HR of 1.16 (95% CI 1.09–1.24). If the QT interval was instead considered a continuous variable, each 10 ms prolongation was associated with an HR of 1.17 (95% CI 1.09–1.25).

#### 3.2.3. QRS Duration

**Figure 3:**
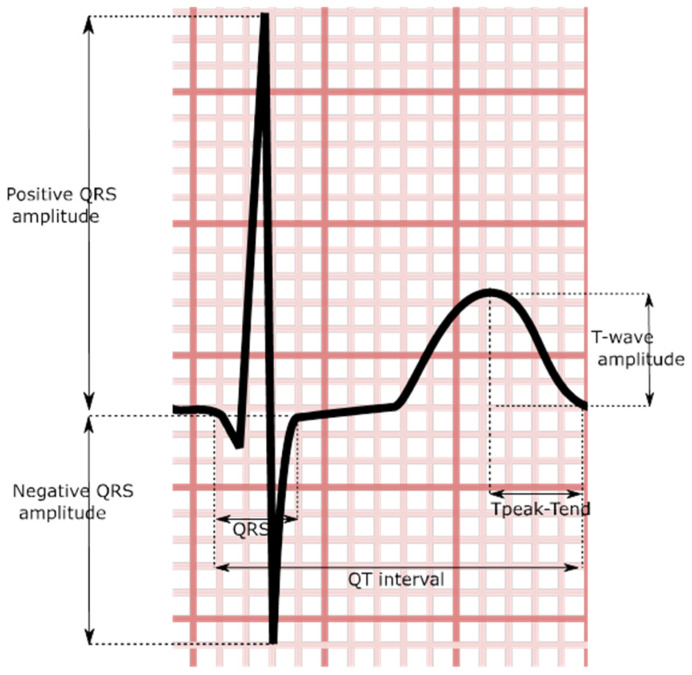
Stylized QRS and t-wave demonstrating certain ventricular indices.

The QRS duration is a simple-to-measure electrocardiographic parameter defined by the duration of time between the start of the QRS complex and the end (Figure 3). With QRS duration prolongation being associated with structural heart disease, it has been suggested that it may act as a proxy for left atrial disease [115].

Seven studies have investigated the role of prolonged QRS duration as a predictor of AF. Several of these were large cohort studies. The results are somewhat variable, with four studies suggesting a minor predictive role for QRS prolongation, either as a continuous variable or with dichotomous cut offs.

In the two studies looking specifically at prediction within stroke populations, the HRs were 1.02 and 1.01. Studies utilized sporadic 12-lead ECGs and patient records for the detection of AF development.

#### 3.2.4. Bundle Brunch Block (BBB)

Bundle branch block (BBB) is a marker of conduction disease. Autopsy reports have shown that conduction disease is due to fibrosis in the conduction system [123], which could be associated with myocardial fibrosis and might explain the rationale behind the association between AF and BBB. There is a degree of overlap between conduction disease and QRS duration prolongation. A variety of studies have looked at the presence of right (RBBB) and left bundle branch block (LBBB), left anterior fascicular block (LAFB) and non-specific interventricular conduction delay (NIVCD).

There have been six studies that look at a variety of different manifestations of conduction disease.

Only one of the three LBBB studies demonstrated a positive predictive relationship for AF. Neither of the RBBB studies suggested any association with future AF; however, interestingly, Frontera’s et al., 2015 study suggested a strong relationship between the presence of incomplete RBBB and future AF. Two studies have suggested roles for LAFB and NIVCD in AF detection.

#### 3.2.5. QRS Fragmentation

Fragmented QRS (fQRS) is defined as the presence of various RSR patterns with or without q waves on 12-lead ECG. The presence of fQRS in ECG is a sign of delay in ventricular conduction, associated with myocardial scarring, ischemia and fibrosis [124].

Two studies have looked at fQRS in the CKD population and the STEMI population. The former was non-significant, whilst the latter suggested a possible role as a predictor, albeit in a small population.

#### 3.2.6. Poor R-Wave Progression

One study of 5813 patients suggested a positive, but weak association between the presence of poor r-wave progression and AF, with an HR of 1.49 (CI 1.01–2.20).

#### 3.2.7. Frontal QRS-T Angle

The interest in AF predictors has meant slightly more niche ECG parameters have been examined. The frontal QRS-T angle, representing the difference between the QRS and t-wave axis, has gained increasing interest recently as an ECG parameter, although it is not routinely measured by ECG machines. It has been studied in the context of 4282 participants within the CHS, where 1276 participants with an abnormal frontal QRS-T angle were shown to have an HR of 1.55 (95% CI 1.23–1.97) for the development of AF [118].

#### 3.2.8. ST Segment—T-Wave Abnormalities

ST-T changes have also been linked to AF. ST segment abnormalities may reflect underlying myocardial changes, including hypertrophy and or/overload that can cause AF, but not severe enough to precipitate other cardiac diseases [108].

Four large cohort studies all demonstrated a statistically significant association between a variety of ST segment and t-wave abnormalities. One of the studies utilized the Minnesota criteria to objectively define ST segment and t-wave abnormalities (Supplementary Table S3). None of the studies utilized continuous methods of rhythm monitoring, instead relying on sporadic follow-up ECGs or retrospective assessment of medical records.

The Tpeak-Tend interval as a specific component of the ventricular repolarization waveform has also been assessed within the Copenhagen ECG study. Here, a U-shaped relationship between the parameter and detection of AF was suggested with values outside of 98–103 ms with an HR of 1.18 (95% CI 1.06–1.32) for the development of AF [119].

## 4. Discussion

### 4.1. Summary of Findings

This study fills an important gap in the current literature. There have been two previous review articles of ECG predictors of AF [13,125], the most recent of which was in 2017. Neither of these studies were systematic in their approach to identifying relevant studies, and they specifically focused on large population studies. This study provides a comprehensive analysis of the current state of the field, with consideration of smaller studies of at-risk or important populations, such as individuals who have hypertrophic cardiomyopathy or stroke.

**Figure 4:**
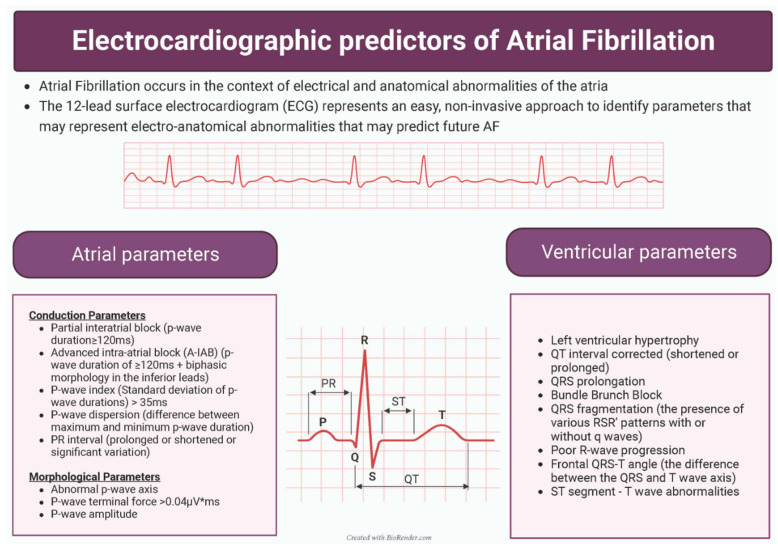
Summary of identified electrocardiographic predictors of atrial fibrillation.

When considering the utility of individual parameters as predictors for AF, the combination of ease of calculation, reliability and strength as a predictor are all important facets. Figure 4 provides a summary of the identified predictors. The present review has highlighted that atrial parameters are particularly useful, and there exists a reasonable amount of evidence for A-IAB, PWTFV1 and PWD as being useful AF predictors. All of these predictors require further assessment of the ECG beyond the numerical values that are calculated. P-wave axis and p-wave amplitude have both shown consistent promising results, but in a limited number of studies. Ventricular parameters were generally not as useful as predictors. Indeed, it is not clear if the predictive power of the ventricular parameters is wholly independent of the atrial parameters.

As alluded to by Smith et al., there is an overlap between components (different components of p-wave) [25]. Disentangling this overlap is important as it facilitates a greater understanding of the parameters that are most useful as AF predictors and potentially provides understanding regarding the mechanistic reasons as to why these parameters are useful.

The reproducibility of measurements both at a single time point and across a period of time has not been examined fully. Composite measures, such as PTFV1, have been critiqued as being particularly susceptible to lead position variation.

Table 1, Table 2 and Table 3 all demonstrate that there are a multitude of different approaches used across studies to detect AF. The most common approaches are ad hoc ECGs and Holter monitors, as well as retrospective assessment of patient notes, registry data and death certificates. These approaches have obvious limitations. The former risks missing paroxysms between recordings, whilst the latter is limited by the accuracy of coding, as demonstrated by Shah et al [126].

A limited number of studies have utilized device EGMs, which have the advantage of providing a continuous rhythm recording from the point of device implantation, and the rise of ILRs has furthered interest in this. Of note, the cut off duration for diagnosing AF was variable across these studies.

### 4.2. The Logistics of AF Prediction

One unstudied aspect of ECG prediction of AF is the temporal evolution of ECG parameters. It is not clear if it is the change in a parameter or the absolute value of the parameter that is critical in the development of AF. Longitudinal studies would be useful here as it would be possible to evaluate the pattern of change in a parameter (if it exists) as a predictor of AF.

The digitization of patient records and ECGs has created a particularly rich data resource. Hospital-wide ECG analysis programs already exist, whereby any individual who undergoes an ECG is specifically screened for AF. With the advent of machine learning and artificial intelligence, more sophisticated screening approaches could be used, utilizing some of the above identified parameters, mainly p-wave indices, to help identify patients at risk of developing AF at the earliest possible stage.

### 4.3. The Role of AF Prediction

AF is endemic within the older population and is associated with significant morbidity and mortality. Its early prediction could offer several possible avenues for further management.

If it is not possible to prevent the development of AF, avoiding its consequences, including stroke, would also be of significant interest. Given the simplicity of administration and improved safety profile of direct oral anticoagulants, targeted use of anticoagulation in high-risk groups could potentially help reduce the incidence of stroke. Indeed, within the ESUS group, AF prediction could be used to target those patients who would benefit most from targeted longer-term cardiac monitoring approaches, or empirical anticoagulation.

### 4.4. Multi Dimension Risk Prediction

Combining ECG parameters may help to maximize AF prediction. This was neatly demonstrated by both Alexander et al. and Yoshizawa et al [34,127]. The former used a morphology–voltage–p-wave duration-based risk model, which had an OR of 2.1 and 2.4 for the intermediate and high-risk groups, respectively, based on a cohort of 676 patients undergoing coronary angiography. The latter used a p-wave amplitude in II and V1 and a p-wave dispersion-based score, with less promising results.

Of course, the 12-lead ECG is not the only parameter which provides data for AF risk. Much work has been conducted on biochemical, Holter, clinical and echocardiographic parameters to aid in AF prediction [68,128,129,130]. Creating a multi-dimension model of risk prediction would provide a more holistic and hopefully accurate model for stratifying AF risk. This could be valuable in the stroke population not only in targeting populations that may benefit the most from invasive monitoring, but also creating stroke primary prevention strategies.

### 4.5. The Role of Artificial Intelligence and Consumer-Facing Devices in AF Prediction

The rise of artificial intelligence (AI) technologies and consumer-facing wearable devices are providing exciting new avenues for AF prediction. Groups from America [131] and Sweden [132] have created machine learning algorithms for the prediction of AF based on a 12-lead ECG and a single-lead ECG, respectively. AI-based models have been shown to have a comparative performance to conventional risk scores, such as the CHARGE-AF score, without the need for significant data extraction [133]. The utilization of feature visualization techniques has yielded analysis of AI-based algorithms to identify which areas the algorithms focus on for AF prediction. Unsurprisingly, algorithms appear to focus on the p-wave for AF prediction, although there also appears to be a contribution from the initial component of the QRS complex [134]. The primary limitation of AI-based algorithms, similar to any AF prediction approach, remains the provenance of the data input and the approach to AF identification. Highly curated ILR-based datasets remain uncommon, with AF diagnoses for training datasets usually based on medical record analysis. Moreover, input data require individuals to have had an ECG at some point, and thus they may not provide a full representation of a general population.

Consumer-facing wearable devices have provided the potential for data from wider cohorts to be assessed, as well as for longitudinal analyses to be performed. Whilst not applied to AF as of yet, the Mayo group have demonstrated the utility of AI assessment of smart watch data to predict left ventricular dysfunction [135]. Algorithms that can work across the different modalities of consumer-facing devices will be of particular use, given the growing number of devices that are available to both consumers and physicians.

This does raise the question as to whether there remains a role for conventional analysis of ECG parameters. As mentioned, identification of key ECG parameters that predict future AF may help facilitate improved understanding of the pathogenesis of AF, and this process may be aided by feature visualization of AI algorithms.

## 5. Conclusions

We have systematically reviewed evidence for the use of different surface ECG parameters as predictors for AF. This is an area of increasing interest, with several parameters showing association with pAF. More work is required to help refine these parameters and the relative predictive risk to each other, to understand their pathophysiological basis in the development of AF and to maximize their use in identifying this group of patients early, particularly in combination with other variables.

## Figures and Tables

**Table 1:** Atrial conduction parameters predictive of atrial fibrillation.

Authors, Year	Population (Size)	Study Type	Parameter Definition	Result	AF Detection
***P-wave duration and Partial Interatrial Block (P-IAB)***					
Kreimer et al., 2021 [14]	Patients undergoing ILR for syncope, palpitations, ESUS (366)	Retrospective	Presence of P-IAB (p-wave ≥ 120 ms)	NS in multivariable analysis	ILR AF ≥ 30 s
Rasmussen et al., 2020 [15]	General population, 55–75 years (632)	Retrospective	P-wave duration II > 120 ms	HR 1.81 (95% CI 0.95–3.45)	ECG, inpatient monitoring
Istolahti et al., 2020 [16]	Finnish adults > 30 years (6354)	Retrospective	Presence of P-IAB (p-wave ≥ 120 ms)	HR 1.39 (95% CI 1.09–1.77)	Medical records (ICD codes) or medications for AF
Hellman et al., 2020 [17]	CKD stage 4–5 (165)	Prospective	P-wave duration (lead II)	*p* = 0.79	ECG, 24 h Holter
Edenborn et al., 2019 [18]	HFpEF (99)	Prospective	Amplified p-wave > 175 ms	HR 9.68 (95% CI 2.61–35.89)	ECG, Holter
Acampa et al., 2018 [19]	ESUS (222)	Prospective	P-wave duration (max)	OR 1.01 (95% CI 0.99–1.03)	7-day ECG monitor
Skov et al., 2018 [20]	Primary care patients, 50–90 years (152,759)	Retrospective	Presence of P-IAB (median p-wave ≥ 120 ms)	HR 1.25 (95% CI 1.19–1.30)	Medical records
Lehtonen et al., 2018 [21]	Hypertensive (2665) Non-hypertensive (3148) (5813)	Prospective	Presence of P-IAB (p-wave max in any lead ≥ 120 ms)	HR 1.36 (95% CI 1.05–1.76)	ICD codes
Roessel et al., 2017 [22]	Italian registry, 25–79 years (240)	Retrospective	P-wave 110–119 ms P-wave 120–129 ms P-wave ≥ 130 ms	OR 5.33 (95% CI 1.74–16.33) OR 5.08 (95% CI 1.73–14.90) OR 5.44 (95% CI 1.95–15.15) Vs p-wave duration < 110 ms	ECG
Alexander et al., 2017 [23]	NSTEMI (322)	Retrospective	Presence of P-IAB	*p* = 0.144	Medical records
Conte et al., 2017 [24]	Patients with AF (36) and healthy control subjects (40) (76)	Retrospective case control	Prolonged p-wave ≥ 125 ms (lead II)	Patients with history of AF had longer p-wave duration (125 ± 18 vs. 110 ± 8 ms, *p* < 0.001)	ECG, Holter
Smith et al., 2017 [25]	ARIC (14,924)	Prospective	Prolonged p-wave (max) > 95th percentile of their distribution	HR 1.48 (95% CI 1.26–1.75)	ECG, medical records, death certificates
Cortez et al., 2017 [26]	Ischemic stroke patients from LSR (227)	Prospective	P-wave duration	HR 1.02 (95% CI 0.96–1.05)	ECG
Cinier et al., 2016 [27]	STEMI patients (198)	Prospective observational	Presence of P-IAB (p-wave ≥ 120 ms)	OR 5.10 (95% CI 1.46–17.80)	ECG, Holter
Wu et al., 2016 [28]	Hospitalized patients (1571)	Prospective	Presence of P-IAB (p-wave ≥ 120 ms)	HR 8.66 (95% CI 5.27–14.23)	Medical records
Magnani et al., 2015 [29]	FHS and ARIC participants (113,64)	Prospective	Prolonged p-wave > 120 ms	HR 1.55 (95% CI 1.29–1.85)	ECG, Holter (FHS) ICD codes (ARIC)
Nielsen et al., 2015 [30]	Copenhagen ECG study (285,933)	Prospective	P-wave ≤ 89 ms P-wave 112–119 ms P-wave 120–129 ms P-wave ≥ 130 ms	HR 1.60 (95% CI 1.41–1.81) HR 1.22 (95% CI 1.13–1.31) HR 1.50 (95% CI 1.39–1.62) HR 2.06 (95% CI 1.89–2.23)	Medical records
Francia et al., 2015 [31]	Hypertensive patients (88)	Retrospective case control	P-wave ≥ 100 ms (lead aVR)	RR 3.70 (95% CI 1.30–10.30)	Case-control study (44 patients with AF and 44 without AF)
Hayashi et al., 2015 [32]	Patients with biphasic p-wave in lead II (141)	Retrospective	Duration of the initial portion of p-wave (lead III) ≥ 71 ms	HR 2.90 (95% CI 1.16–7.11)	ECG
Chang et al., 2014 [33]	Patients with lone AF (<60 years and no risk factors for AF) (61) and controls without AF (150) (211)	Retrospective case control	Shorter p-wave duration (min) For one tetrile increment (p-wave duration min) For one tetrile increment (p-wave duration max) P-wave duration (min) < 69 ms	OR 0.63 (95% CI 0.42–0.93) OR 0.95 (95% CI 0.63- 1.43) Separated patients with paroxysmal lone AF from healthy controls with a sensitivity of 70%, specificity of 48%	Case-control study (61 patients with AF and 150 without AF)
Yoshizawa et al., 2014 [34]	Patient with (68) and without AF (68) (132)	Case control	P-wave duration II P-wave duration V1	Similar between patients with AF versus those without (87.6 ± 20.4 vs. 86.3 ± 17.5 ms, *p* = 0.702) Similar between patients with AF versus those without (78.5 ± 24.8 vs. 76.8 ± 16.7 ms, *p* = 0.628)	ECG
Girasis et al., 2013 [35]	HCM with AF (30) and sex- and age-matched controls without AF (32) Sex- and age-matched healthy individuals (25) (62)	Retrospective case control	P-wave duration in Z-lead (orthogonal ECG)	OR 1.08 (95% CI 1.02–1.14)	Case control
Dogan et al., 2011 [36]	Acute ischemic stroke (400)	Retrospective	P-wave duration (max) (per 10 ms increase)	OR 1.11 (95% CI 0.68–1.83)	Holter AF ≥ 30 s
Magnani et al., 2011 [37]	FHS participants ≥ 60 years (1550)	Prospective	Upper 5% of max p-wave	HR 2.51 (95% CI 1.13–5.57)	ECG
Radeljic et al., 2011 [38]	PPM for CHB > 70 years (81)	Prospective	P-wave > 100 ms	OR 16.5 (95% CI 2.97–91.69)	Device EGM AHRE > 5 min
Macfarlane et al., 2011 [39]	PROSPER study participants aged 70–82 years (5804)	Prospective	P-wave duration (per 20 ms increase)	HR 1.08 (95% CI 0.96–1.20)	ECG
Soliman et al. 2009 [40]	ARIC participants (15,429)	Prospective	Mean p-wave (per 1 SD change) Max p-wave (per 1 SD change) P-wave lead II (per 1 SD change) Mean p-wave (upper 5th percentile) Max p-wave (upper 5th percentile) P-wave lead II (upper 5th percentile)	HR 1.64 (95% CI 1.34–2.00) HR 1.79 (95% CI 1.51–2.14) HR 1.80 (95% CI 1.49–2.20) HR 3.21 (95% CI 1.93–5.31) HR 4.07 (95% CI 2.255–6.51) HR 3.90 (95% CI 2.42–6.27)	ECG
Perez et al., 2009 [41]	Patients that had an ECG for usual indications (42,751)	Retrospective	P-wave max > 120 ms	HR 1.60 (95% CI 1.30–1.80)	ECG
Ariyarajah et al., 2007 [42]	Patients with comparable echocardiographic parameters (32)	Prospective	Presence of P-IAB (p-wave max ≥ 120 ms)	HR 6.70 (95% CI 1.04–42.8)	Medical records
Ozdemir et al., 2005 [43]	HCM Patients with AF (27) and age-matched healthy control subjects (53) (80)	Prospective case control	P-wave max > 134.5 ms	RR 9.9 (95% CI 18.3–521)	ECG, Holter
Aras et al., 2005 [44]	Hyperthyroidism (133)	Retrospective case control	P-wave duration (max)	*p* = 0.03	Case control
Kristensen et al., 2004 [45]	PPM for SND (109)	Retrospective	P-wave duration	NS difference in patients with (107 ± 16) and without AF (105 ± 13)	ECG Device ECG (AHRE with a rate of ≥220 bpm lasting for ≥5 min, mode switching ≥5% of total time recorded or a mode switching episode lasting ≥5 min)
Altunkeser et al., 2003 [46]	Patients with structural heart disease and LAD ≤ 5.0 cm with AF (37) and without AF (38) (75)	Case control	P-wave duration (max) P-wave duration (max) ≥ 110 ms	*p* < 0.05 Separated patients with AF and controls with a sensitivity of 80%, specificity of 52% and positive predictive accuracy of 62%	Case-control study
Köse et al., 2003 [47]	HCM patients with AF (22) and without AF (26) (48)	Retrospective case control	P-wave (max) P-wave (min)	134 ± 11 vs. 128 ± 13 ms, *p* = 0.06 78 ± 9 vs. 81 ± 7 ms, *p* = 0.07	Case-control study
Aytemir et al., 2000 [48]	Patients with AF (90) and healthy control subjects (70) (160)	Case control	P-wave (max) > 106 ms	Separated patients with AF and controls with a sensitivity of 83%, specificity of 72% and positive predictive accuracy of 79%	Case-control study
Ozer et al., 2000 [49]	Hypertensive patients with AF (44) and without AF (50) (94)	Retrospective case control	P-wave (max) P-wave (min)	NS in multivariable analysis *p* = 0.60 in univariable analysis	ECG
Dilaveris et al., 1998 [50]	Patients with AF (60) and age-matched healthy control subjects (40) (100)	Retrospective case control	P-wave ≥ 110 ms	*p* < 0.0001 Sensitivity of 88%, specificity of 75%, positive predictive accuracy of 84%	Case-control study
***Advanced interatrial block (A-IAB): p-wave duration > 120 ms + biphasic inferior p-wave morphology in the inferior leads***					
Kreimer et al., 2021 [14]	Patients undergoing ILR for syncope, palpitations, ESUS (366)	Retrospective	Presence of A-IAB (p-wave duration max in any lead)	HR 5.01 (95% CI 2.64–9.53)	ILR AF ≥30 s
Istolahti et al., 2020 [16]	Finnish adults >30 years (6354)	Retrospective	Presence of A-IAB	HR 1.63 (95% CI 1.00–2.65)	Medical records (ICD codes) or medications for AF
Hellman et al., 2020 [17]	CKD stage 4–5 (165)	Prospective	Presence of A-IAB (p-wave II ≥ 120 ms and one or more biphasic p-waves in the inferior leads)	*p* = 0.84	ECG, 24 h Holter
Mendieta et al., 2020 [51]	ESUS (75)	Prospective	Presence of A-IAB	*p* = 0.042	Medical records, ECG, Holter
Boccanelli et al., 2019 [52]	PREDICTOR study, 65–84 years (1626)	Prospective	Presence of A-IAB or P-IAB	HR 3.05 (95% CI 1.51–6.18)	Medical records (ICD codes)
Skov et al., 2018 [20]	Primary care patients, 50–90 years (152,759)	Retrospective	Presence of A-IAB	HR 3.38 (95% CI 2.99–3.81)	Medical records
Alexander et al., 2018 [53]	Patients with carotid and coronary disease (355)	Retrospective	Presence of A-IAB or P-IAB	OR 2.40 (95% CI 1.33–4.29)	Medical records, ECG, Holter
Escobar-Robledo et al., 2018 [54]	Chronic HF (464)	Prospective observational	Presence of A-IAB	HR 2.71 (95% CI 1.61–4.56)	Medical records
Roessel et al., 2017 [22]	Italian Registry 25–79 years (240)	Retrospective	Presence of A-IAB	OR 2.09 (95% CI 0.78–5.64)	ECG
Russo et al., 2018 [55]	Myotonic dystrophy type I undergoing PPM (70)	Prospective	Presence of A-IAB or P-IAB	HR 10.76 (95% CI 3.46–33.49)	Device EGM AHRE >200 bpm and lasting >5 min
Tekkesin et al., 2017 [56]	PPM for SND (367)	Prospective	Presence of A-IAB or P-IAB	*p* < 0.01	Device EGM AHRE >5 min and >220 bpm
Alexander et al., 2017 [23]	NSTEMI (322)	Retrospective	Presence of A-IAB or P-IAB	*p* = 0.021	Medical records
O’Neal et al., 2016 [57]	ARIC (14,625)	Prospective	Presence of A-IAB	HR 3.09 (95% CI 2.51–3.79)	ECG, medical records
Ali et al., 2015 [58]	CCF undergoing CRT (97)	Retrospective	Presence of A-IAB	OR 4.13 (95% CI 1.60–10.70)	Device EGM (AHRE ≥ 30 s)
Bayes de Luna et al., 1988 [59]	Patients with A-IAB and controls (32)	Prospective	Presence of A-IAB	93.7% developed paroxysmal supraventricular tachyarrhythmia, *p* < 0.001	Holter
***P-wave index: standard deviation of p-wave durations (across the 12 leads)***					
Perez et al., 2009 [41]	Patients that had an ECG for usual indications (42,751)	Retrospective	P-wave index > 35 ms	HR 1.70 (95% CI 1.30–2.10)	ECG
***P-wave onset to P-wave peak: time between onset of p-wave to peak of p-wave***					
Oz et al., 2020 [60]	ESUS (90)	Retrospective	P-wave onset to p-wave peak (lead II) P-wave onset to p-wave peak (lead V1)	OR 1.34 (95% CI 1.15–1.56) OR 1.12 (95% CI 1.02–1.22)	ECG, Holter
Smith et al., 2017 [25]	ARIC (14,924)	Prospective	Prolonged p-wave onset to p-wave peak (max) > 95th percentile of their distribution	HR 1.57 (95% CI 1.31–1.88)	ECG, medical records
***P-wave peak to p-wave end—time between peak of p-wave to end of p-wave***					
Smith et al., 2017 [25]	ARIC (14,924)	Prospective	Prolonged p-wave peak to p-wave end (max) > 95th percentile of their distribution	HR 1.20 (95% CI 0.99–1.46)	ECG, medical records
***P-wave dispersion (PWD): difference between maximal and minimal p-wave durations***					
Acampa et al., 2018 [19]	ESUS (222)	Prospective	PWD (per 10 ms increase)	OR 1.92 (95% CI 1.45–2.55)	7-day ECG monitor
Yesin et al., 2018 [61]	STEMI patients (171)	Prospective	PWD	OR 1.02 (95% CI 1.01–1.03)	Inpatient monitoring
Rago et al., 2017 [62]	Beta thalassemia major (80)	Prospective	PWD	HR 1.32 (95% CI 0.76–4.82)	30-day ELR performed every 6 months for 5 years (AF > 15 s)
Tuluce et al., 2016 [63]	HCM (70)	Prospective	PWD PWD ≥ 47.5 ms	OR 1.08 (95% CI 1.01–1.15) Predicted AF with sensitivity of 78% and specificity 72%	ECG, 48-h Holter
Chang et al., 2014 [33]	Patients with lone AF (<60 years and no risk factors for AF) (61) and controls without AF (150) (211)	Retrospective case control	PWD For one tertile increment (p-wave duration min)	OR 1.47 (95% CI 0.63–1.43)	Case-control study
Yoshizawa et al., 2014 [34]	Patient with (68) and without AF (68) (132)	Retrospective case control	PWD	OR 1.11 (95% CI 10.07–1.17)	ECG
Dogan et al., 2011 [36]	Acute ischemic stroke (400)	Retrospective	PWD (per 10 ms increase) PWD > 57.5 ms	OR 2.74 (95% CI 1.48–5.07) Predicted AF with a sensitivity of 80%, specificity of 73%, positive predictive value 74% and negative predictive value 78%	Holter AF ≥ 30 s
Perez et al., 2009 [41]	Patients that had an ECG for usual indications (42,751)	Retrospective	PWD > 80 ms	HR 1.95 (95% CI 1.70–2.30) Only when adjusted for age and sex, but not multivariable	ECG
Aras et al., 2005 [44]	Hyperthyroidism (133)	Retrospective case control	PWD	*p* = 0.001	Case-control study
Ozdemir et al., 2005 [43]	HCM Patients with AF (27) and age-matched healthy control subjects (53) (80)	Prospective case control	PWD > 52.5 ms	RR 24 (95% CI 27.6–2251.3)	ECG, Holter
Kristensen et al., 2004 [45]	PPM for SND (109)	Retrospective	PWD	NS difference in patients with (67 ± 22) and without AF (64 ± 18)	ECG Device ECG (AHRE with a rate of ≥220 bpm lasting for ≥5 min, mode switching ≥5% of total time recorded or a mode switching episode lasting ≥5 min)
Altunkeser et al., 2003 [46]	Patients with structural heart disease and LAD ≤5.0 cm with AF (37) and without AF (38) (75)	Case control	PWD	NS in multivariable analysis	Case-control study
Köse et al., 2003 [47]	HCM patients with AF (22) and without AF (26) (48)	Retrospective case control	PWD	55 ± 6 ms vs. 37 ± 8 ms, *p* < 0.001	Case-control study
Tükek et al., 2002 [64]	COPD (40)	Retrospective	PWD	OR 1.36 (95% CI 1.01–1.83)	Medical records, Holter
Aytemir et al., 2000 [48]	Patients with AF (90) and healthy control subjects (70) (160)	Case control	PWD > 36 ms	Separated patients with AF and controls with a sensitivity of 77%, specificity of 82% and positive predictive accuracy of 85%	Case-control study
Ozer et al., 2000 [49]	Hypertensive patients with AF (44) and without AF (50) (94)	Retrospective case control	PWD	<0.001	ECG
Dilaveris et al., 1998 [50]	Patients with AF (60) and age-matched healthy control subjects (40) (100)	Retrospective case control	PWD ≥ 40 ms	*p* < 0.0001 Sensitivity of 83%, specificity of 85%, positive predictive accuracy 89%	Case-control study
***P-wave dispersion (PWD)—p-wave duration/Pvm***					
Cortez et al., 2017 [26]	Ischemic stroke patients from LSR (227)	Prospective	PWD	HR 2.02 (95% CI 1.00–1.02)	ECG
*PQ interval*					
Cortez et al., 2017 [26]	Ischemic stroke patients from LSR (227)	Prospective	PQ interval	HR 1.00 (95% CI 0.99–1.01)	ECG
Hayashi et al., 2014 [65]	Patients with p-pulmonale (591)	Retrospective	PQ interval > 150 ms	HR 6.89 (95% CI 2.39–29.15)	ECG
***PR segment: time between end of p-wave and start of QRS complex (maximum PR interval: maximum p-wave duration)***					
Smith et al., 2017 [25]	ARIC (14,924)	Prospective	Prolonged PR segment 9 max) >95th percentile of their distribution	HR 1.05 (95% CI 0.85–1.29)	ECG, medical records, death certificates
*Prolonged PR interval*					
Kreimer et al., 2021 [14]	Patients undergoing ILR for syncope, palpitations, ESUS (366)	Retrospective	PR interval	NS in multivariable analysis	ILR AF ≥ 30 s
Hellman et al., 2020 [17]	CKD stage 4–5 (165)	Prospective	PR interval (lead II)	*p* = 0.48	ECG, 24 h Holter
Lehtonen et al., 2018 [21]	Hypertensive (2665) Non-hypertensive (3148) (5813)	Prospective	Prolonged PR interval ≥ 220 ms	HR 1.67 (95% CI 1.16–2.41)	ICD codes
Acampa et al., 2018 [19]	ESUS (222)	Prospective	PR interval	OR 1.00 (95% CI 0.99–1.01)	7-day ECG monitor
Conte et al., 2017 [24]	Patients with AF (36) and healthy control subjects (40) (76)	Retrospective case control	PR interval	Similar between patients with and without AF (*p* = 0.57)	ECG, Holter
Smith et al., 2017 [25]	ARIC (14,924)	Prospective	Prolonged PR interval (max) > 200 ms and PR interval >95th percentile of their distribution	HR 1.19 (95% CI 1.02–1.40)	ECG, medical records, death certificates
Chun et al., 2016 [66]	Patients with frequent SVEs (>100 SVEs/day) (684)	Retrospective	Prolonged PR interval > 200 ms	HR 1.95 (95% CI 1.03–3.70)	ECG, Holter
Chun et al., 2016 [67]	Patients with frequent SVEs (>100 SVEs/day) (207)	Retrospective	Prolonged PR interval > 200 ms PR variation (PR interval max-PR interval min)	HR 3.32 (95% CI 1.06–10.36) HR 1.01 (95% CI 1.00–1.02)	ECG, Holter
Cabrera et al., 2016 [68]	Patients undergoing Holter for any cause (299)	Retrospective	Increasing PR interval	HR 1.01 (95% CI 1.00–1.02)	Medical records, ECG, Holter, Device EGM, ILR showing AF lasting ≥30 s
Thijs et al., 2016 [69]	ESUS (CRYSTAL AF-ILR arm) (221)	Prospective	Increasing PR interval (per 10 ms increase)	HR 1.30 (95% CI 1.20–1.40)	ILR AF lasting ≥30 s
Hayashi et al., 2015 [32]	Patients with biphasic p-wave in lead II (141)	Retrospective	PR interval	PR interval similar between patients with AF versus those without (184.1 ± 40.3 vs. 170.8 ± 44.5 ms, *p* = 0.15)	ECG
Shulman et al., 2015 [70]	African American, Hispanic and non-Hispanic white (50,870)	Retrospective	PR interval (per 10 ms increase) PR interval 196–201 ms (Hispanic and African Americans) PR interval 203–212 ms (non- Hispanic Whites)	HR 1.04 (95% CI 1.03–1.05) HR 1.42 (95% CI 1.09–1.86) HR 1.32 (95% CI 1.07–1.64)	ECG
Frontera et al., 2015 [71]	Patients undergoing ILR implant for syncope or palpitations (200)	Retrospective	PR interval	OR 1.14 (95% CI 0.69–1.89)	ILR AF lasting > 30 s
Aro et al., 2014 [72]	Individuals 30–59 years old (10,785)	Prospective	Prolonged PR interval (longest in the bipolar limb) > 200 ms	HR 1.03 (95% CI 0.74–1.45)	Medical records, ECG
Knuiman et al., 2014 [73]	Busselton Health Study participants (4267)	Prospective	Short PR interval < 120 ms Long PR interval ≥ 220 ms	HR 6.21 (95% CI 1.52–25.31) HR 1.29 (95% CI 0.68–2.44)	ICD codes
Magnani et al., 2013 [74]	Health ABC study (2722)	Prospective	Prolonged PR interval (lead II) PR > 200 ms Per 1 SD increase (29 ms) in PR interval	HR 1.26 (95% CI 0.99–1.61) HR 1.13 (95% CI 1.04–1.23)	ICD codes
Nielsen et al., 2013 [75]	Copenhagen ECG study (288,181)	Retrospective	Prolonged PR interval (distance between the earliest detection of atrial and ventricular depolarization in any lead) (median from 12 leads) PR interval ≥ 196 ms women PR interval ≥ 204 ms men Shorter PR interval (median from 12 leads) PR interval ≤ 121 ms women PR interval ≤ 129 ms men	HR 1.18 (95% CI 1.06–1.30) HR 1.30 (95% CI 1.17–1.44) HR 1.32 (95% CI 1.12–1.56) HR 1.09 (95% CI 0.92–1.29)	Medical records
Macfarlane et al., 2011 [39]	PROSPER study, 70–82 year old participants (5804)	Prospective	PR prolongation (per 30 ms increase)	HR 1.29 (95% CI 1.29–1.41)	ECG
Soliman et al., 2009 [40]	ARIC participants (15,429)	Prospective	PR duration (per 1 SD change) PR duration (upper 5th percentile) (mean p-wave duration + mean PR segment duration)	HR 1.41 (1.20–1.65) HR 1.59 (0.77–3.30)	ECG
Perez et al., 2009 [41]	Patients that had an ECG for usual indications (42,751)	Retrospective	Prolonged PR interval > 200 ms	HR 1.30 (95% CI 1.10–1.60)	ECG
Cheng et al., 2009 [76]	FHS participants (7575)	Prospective	Prolonged PR interval > 200 ms (lead II)	HR 2.06 (95% CI 1.36–3.12)	Medical records, ECG

AF, atrial fibrillation; AHRE, atrial high rate episodes; A-IAB, advanced interatrial block; ARIC, atherosclerosis risk in communities; bpm, beats per minute; CCF, congestive heart failure; CHB, complete heart block; CI, confidence interval; cm, centimeter; CKD, chronic kidney disease; COPD, chronic obstructive pulmonary disease; CRT, cardiac resynchronization therapy; CRYSTAL AF, cryptogenic stroke and underlying AF; DM1, myotonic dystrophy type 1; ECG, electrocardiogram; EGM, intracardiac electrogram; ELR, externalized loop recorder; ESUS, embolic stroke of undetermined source; FHS, Framingham Heart Study; HCM, hypertrophic cardiomyopathy; Health ABC, The Health Aging and Body Composition; HF, heart failure; HFpEF, heart failure with preserved ejection fraction; HR, hazard ratio; ICD, international classification of diseases; ILR, implantable loop recorder; LAD, left atrial diameter; LSR, Lund Stroke Register; min, minute; ms, milliseconds; NS, non-significant; NSTEMI, non-ST-elevation myocardial infarction; OR, odds ratio; P-IAB, partial interatrial block; PPM, permanent pacemaker; PROSPER study, PROspective Study of Pravastatin in the Elderly at Risk; Pvm, p-wave vector magnitude; PWD, p-wave dispersion; RR, relative risk; s, second; SD, standard deviation; SND, sinus node disease; STEMI, ST-elevation myocardial infarction; SVE, supraventricular ectopic.

## Data Availability

Not applicable.

## Supplementary Materials

The following supporting information can be downloaded at: https://www.mdpi.com/article/10.3390/medsci11020030/s1, Table S1: ECG criteria for LVH; Table S2: QT correction formulae, Table S3: Minnesota code ST-segment abnormalities [136,137,138,139,140,141,142,143].
