# Supplementary material for: Electrocardiographic Predictors of Atrial Fibrillation
Source: Med Sci (Basel). 2023 Apr 7;11(2):30. doi: 10.3390/medsci11020030 (PMC10123668; doi:10.3390/medsci11020030)
Supplement: Supplementary file 1 [file medsci-11-00030-s001.zip › medsci-2302940-supplementary.pdf]

Electrocardiographic Predictors of Atrial Fibrillation

Panagiota Anna Chousou MD, MRCP(UK), ECDS [1,2,#] Rahul Chattopadhyay MBBChir, MRCP(UK) [1,2,#] Vasiliki Tsampasian MD, MSc, MRCP(UK) [1,3] * Vassilios S Vassiliou MA, MBBS, MRCP(UK), PhD, FHEA, FESC,[1,3] * Peter John Pugh MBBS, MD, FRCP, FESC[2]

Supplementary Material

Supplemental Table 1: ECG criteria for LVH	
Criteria	Definition
Sokolow–Lyon[1]	S in V_1 + R in V_5 or V_6 (whichever is larger) ≥ 35 mm (≥ 7 large squares)<br>R in aVL ≥ 11 mm
Romhilt–Estes [2]<br>(based on points: diagnostic >5 points, probable 4 points)	<u>3 points</u><br>• R or S in limb leads ≥20 mm<br>• S in V_1 or V_2 ≥30 mm<br>• R in V_5 or V_6 ≥30 mm<br>• ST-T vector opposite to QRS without digitalis<br>• Negative terminal p mode in V_1 1 mm in depth and 0.04 sec in duration<br><u>2 points</u><br>• Left axis deviation<br><u>1 point</u><br>• ST-T vector opposite to QRS with digitalis<br>• QRS duration ≥0.09s<br>• Delayed intrinsic deflection in V5 or V6 (>0.05s)
Cornell voltage[3]	S in V_3 + R in aVL > 28 mm (men)<br>S in V_3 + R in aVL > 20 mm (women)
Minnesota code[4]	Definite: very high QRS voltage plus ST-T changes<br>Probable: less high though abnormal QRS voltage plus ST-T changes<br>Possible: very high voltage only
Gubner and Ungerleider Voltage	RI + SIII $\geq$ 2.2mV

Supplemental table 2: QT correction formulae	
QT correction	Formula
Bazzett [5]	$QT_c = \frac{QT}{\sqrt{RR}}$
Hodges [6]	$QT_c = QT + 1.75\ (HR - 60)$
Framingham [7]	$QT_c = QT + 0.154\ (1 - RR)$
Fredericia [8]	$QT_c = \frac{QT}{\sqrt[3]{RR}}$

Supplemental table 3: Minnesota code ST-segment abnormalities	
Minnesota Code	ST-segment abnormality
Mild	• Flat T wave (code 5-3 or 5-4)<br>• Negative or biphasic T-wave (negative-positive type) with a negative phase < 1.0 mm (code 5-3)
Severe	• Negative or biphasic T-wave (negative-positive type) with negative phase ≥1.0 mm (code 5-1 or 5-2)<br>• Horizontal or downward sloping ST-segment depression ≥0.5 mm (code 4-1 or 4-2)<br>• Upward sloping ST depression ≥1.0 mm (code 4-4).
